# Complex interactions in a novel *SCN5A* compound mutation associated with long QT and Brugada syndrome: Implications for Na^+^ channel blocking pharmacotherapy for *de novo* conduction disease

**DOI:** 10.1371/journal.pone.0197273

**Published:** 2018-05-23

**Authors:** Jie Liu, Jason D. Bayer, Roozbeh Aschar-Sobbi, Marianne Wauchop, Danna Spears, Michael Gollob, Edward J. Vigmond, Robert Tsushima, Peter H. Backx, Vijay S. Chauhan

**Affiliations:** 1 Department of Biology, York University, Toronto, Ontario, Canada; 2 Electrophysiology and Heart Modeling Institute (LIRYC), Bordeaux University Foundation, Pessac, France; 3 University of Bordeaux, IMB, UMR 5251, Talance, France; 4 Peter Munk Cardiac Center, Division of Cardiology, University Health Network, Toronto, Ontario, Canada; University of Milan, ITALY

## Abstract

**Background:**

The *SCN5A* mutation, P1332L, is linked to a malignant form of congenital long QT syndrome, type 3 (LQT3), and affected patients are highly responsive to the Na^+^ channel blocking drug, mexiletine. In contrast, A647D is an atypical *SCN5A* mutation causing Brugada syndrome. An asymptomatic male with both P1332L and A647D presented with varying P wave/QRS aberrancy and mild QTc prolongation which did not shorten measurably with mexiletine.

**Objective:**

We characterized the biophysical properties of P1332L, A647D and wild-type (WT) Na^+^ channels as well as their combinations in order to understand our proband’s phenotype and to guide mexilitine therapy.

**Methods:**

Na^+^ channel biophysics and mexilitine-binding kinetics were assessed using heterologous expression studies in CHO-K1 cells and human ventricular myocyte modeling.

**Results:**

Compared to WT, P1332L channels displayed a hyperpolarizing shift in inactivation, slower inactivation and prominent late Na^+^ currents (I_Na_). While A647D had no effect on the biophysical properties of I_Na_, it reduced peak and late I_Na_ density when co-expressed with either WT or P1332L. Additionally, while P1332L channels had greater sensitivity to block by mexiletine compared to WT, this was reduced in the presence of A647D. Modelling studies revealed that mixing P1332L with A647D channels, action potential durations were shortened compared to P1332L, while peak I_Na_ was reduced compared to either A647D coexpressing with WT or WT alone.

**Conclusions:**

While A647D mitigates the lethal LQT3 phenotype seen with P1332L, it also reduces mexilitine sensitivity and decreases I_Na_ density. These results explain our proband’s mild repolarization abnormality and prominent conduction defect in the atria and ventricles, but also suggest that expression of P1332L with A647D yields a novel disease phenotype for which mexiletine pharmacotherapy is no longer suitable.

## Introduction

Mutations in *SCN5A* can alter the biophysical properties of the cardiac Na^+^ channel and lead to several different hereditary arrhythmias, including long QT syndrome (LQTS type 3 or LQT3), Brugada syndrome, sick sinus syndrome, heart block and atrial fibrillation (AF) [1]. Although conduction abnormalities have been described with these mutations in the ventricle, sinoatrial node, and atrioventricular (AV) node, the effect on atrial activation has not been previously reported. In some LQT3 *SCN5A* mutations, Na^+^ channel blockers, such as mexiletine, can shorten the QTc interval and reduce the risk of cardiac events. The P1332L LQT3 mutation is particularly virulent causing marked QTc prolongation (>500 ms), AV block and juvenile sudden cardiac death [2–4]. This mutation is highly sensitive to mexiletine and treated patients have reversal of their lethal phenotype [2–4]. In contrast, the atypical A647D *SCN5A* mutation is reported to cause Brugada syndrome without appreciably altering the Na^+^ channel biophysical profile compared to WT [5, 6].

Compound *SCN5A* mutations causing LQT3 are uncommon, and in some instances one mutation rescues the Na^+^ channel abnormalities of the other [7–9]. In these cases, the implications of continuing Na^+^ channel blocking pharmacotherapy have not been investigated. We present a case of a compound *SCN5A* mutation, P1332L and A647D, found in an asymptomatic adult male, who manifested a novel pleiotropic phenotype of mild QTc prolongation, atypical ST elevation and abnormal conduction in both the atrium and ventricle. While mexiletine was initially indicated to treat the P1332L-related LQT3 phenotype, the proband’s resting conduction defects and Brugada-associated A647D mutation caused us to reconsider this treatment strategy [10]. In order to understand the complex phenotype of our proband and to clarify whether mexiletine therapy was suitable, we combined heterologous expression studies of these mutant *SCN5A* channels with computer modeling of ventricular myocyte electrophysiology. In the absence of genetic validation of whether the P1332L and A647D mutations were oriented as *cis* versus *trans*, our expression studies considered both possibilities.

## Materials and methods

### Clinical evaluation

The proband’s clinical evaluation included a standard 12-lead electrocardiogram (ECG), which was recorded at baseline and after intravenous procainamide challenge (10mg/kg infused over 15 minutes). The latter was performed to assess Brugada syndrome. In addition, an invasive electrophysiology study was performed in the fasting state, whereby multielectrode recording catheters were placed percutaneously in the right ventricular apex, high right atrium, His bundle region and coronary sinus. Intracardiac bipolar electrograms (bandpass filter: 30-500Hz, sampling rate: 1000Hz) were recording from these sites during baseline sinus rhythm. The clinical diagnosis was based on this evaluation and performed at University Health Network, Toronto, Canada. The study was approved by the Institutional Review Board of University Health Network and written informed consent was obtained.

### Genetic testing

Genetic testing was performed by an accredited commercial laboratory (Invitae Corp^™^, San Francisco, CA) on a panel of 57 genes implicated in inherited arrhythmias, including LQTS and Brugada syndrome. Genomic DNA was extracted from white blood cells using standard protocols. Next generation sequencing was used to evaluate exons and splice junctions for sequence variants and large deletions or duplications. The following genes were evaluated: ABCC9, ACTN2, AKAP9, ANK2, ANKRD1, CACNA1C, CACNA2D1, CACNB2, CALM1, CALM2, CALM3, CASQ2, CAV3, CTNNA3, DES, DSC2, DSG2, DSP, EMD, GPD1L, HCN4, JUP, KCND3, KCNE1, KCNE2, KCNE3, KCNE5, KCNH2, KCNJ2, KCHJ5, KCNJ8, KCNQ1, LDB3, LMNA, NKX2-5, PDLIM3, PKP2, PLN, PRKAG2, RANGRF, RBM20, RYR2, SCN10A, SCN1B, SCN2B, SCN3B, SCN4B, SCN5A, SLMAP, SNTA1, TGFB3, TMEM43, TNNI3, TNNT2, TRDN, TRPM4, TTN. Control data on variants were obtained from population databases (ExAC).

### Site-directed mutagenesis and heterologous expression

Mutations were introduced into the human *SCN5A* cDNA in the ngPAH1 plasmid using site-directed mutagenesis and verified by full sequencing. The specific mutations introduced into the *SCN5A* cDNA were a A647D mutation, a P1332L mutation, or a combination of the A647D and P1332L mutations. CHO-K1 cells were transfected using lipofectamine 3000 (Life technologies, USA) according to the manufacturer’s protocol. The *SCN5A* cDNA plasmids (1μg) were coexpressed with plasmids containing the GFP-marker plus the β1-subunit cDNA (1μg). When combinations of two *SCN5A* genes were expressed, the total *SCN5A* cDNA contained 0.5μg of each channels type. For example, when wild-type (WT) *SCN5A* was coexpressed with the A647D *SCN5A* mutant (i.e. WT+A647D), we mixed 0.5μg of WT *SCN5A* cDNA plasmid with 0.5μg of A647D *SCN5A* cDNA plasmid.

### Cellular electrophysiology studies

Whole cell Na^+^ currents (I_Na_) in green fluorescent protein-positive CHO-K1 cells, showing green fluorescence under mercury light, were recorded at room temperature using an Axopatch 200B amplifier (Molecular device, USA) under whole-cell patch clamp mode with 80–85% series resistance compensation. The bath solution contained (in mM): 140 NaCl, 4 CsCl, 1 MgCl_2_, 1.2 CaCl_2_, 10 HEPES, 10 D-glucose, pH 7.35 with NaOH. Internal solution contained (in mM): 10 NaCl, 1 CaCl_2_, 135 CsCl, 1 MgCl_2_, 4 MgATP, 10 HEPES, 10 EGTA, pH 7.2 with CsOH. Different mexiletine concentration solutions were prepared by diluting freshly made mexiletine methanol stock (200mM) (Sigma, Mississauga, Canada).

I_Na_ was measured as a function of voltage in response to 500ms depolarizing pulses (V_m_) ranging between -80 to +50mV from a holding potential at -110mV. To assess the activation properties and densities of Na^+^ channel expression, we estimated the whole-cell Na^+^ channel conductance (G_Na_) at various depolarizing voltages (V_m_) by dividing the measured peak I_Na_ (I_Na_^peak^) by the electrochemical driving force V_m_-E_Na_, where E_Na_ is the equilibrium voltage (estimated to be +55mV). To assess the steady state activation and the densities of Na^+^ expression, we plotted G_Na_ as a function of V_m_ and fitted to the Boltzmann equation:

$$G_{Na}^{Peak}=G_{Na}^{Max}/(1+e^{(V_{\frac{1}{2}}^{act}-V_{m})/k})$$ (1)

where G_Na_^Max^ is the maximal conductance, V_1/2_^act^ is the voltage (V_m_) at which the Na^+^ channels are activated by 50% and k is the slope factor.

Steady-state inactivation was determined by applying a family of 500ms conditioning pulses to voltages ranging from -100 to -10mV followed by a 50ms test pulse to 0mV. The holding potential was -110mV which was applied for 4 seconds between each conditioning pulse. To assess steady-state inactivation properties, peak I_Na_ measured during the test pulse to 0mV was normalized to the maximum peak I_Na_ (I_Na_^Max^) and plotted against the conditioning voltage. These data were fit to a Boltzmann equation:

$${Peak\;I_{Na}/I}_{Na}^{Max}=1/(1+e^{(V_{m}-V_{\frac{1}{2}}^{inact})/k})$$ (2)

where V_1/2_^inact^ is the voltage (V_m_) at which the Na^+^ channels are inactivated by 50% and k is the slope factor.

To assess the inactivation kinetics of *SCN5A* channels, we measured the time for 50% decay of I_Na_ from its peak. We also fit the time course of I_Na_ decay to multi-exponential functions using algorithms previously reported [11]. We found that in the majority of CHO-K1 cells, I_Na_ was best fit statistically with a tri-exponential function:

$$f(t)=A_{1}e^{-t/\tau_{1}}+A_{2}e^{-t/\tau_{2}}+A_{3}e^{-t/\tau_{3}}+C$$ (3)

where τ are inactivation time constants, A are the proportion of each component and C is a measure of the non-inactivating (sustained) current. We also assessed inactivation properties of *SCN5A* channels by applying ramp protocols which involved initially activating the I_Na_ by a 5ms step to +20mV from a holding potential of -100mV followed by a 150ms ramp back to -100mV. To eliminate the background leak currents, we applied a P/4 protocol from -120mV 5 seconds prior to the voltage protocol.

Recovery from inactivation of I_Na_ was assessed by applying the standard double pulse protocol wherein channels were held at -100mV after which a depolarizing pulse to -20mV was applied for 50ms (i.e. the conditioning pulse) which ensures complete inactivation. The membrane was then repolarized back to -100mV for variable periods of time ranging from 10ms to several seconds, followed by a second step to -20mV (i.e. the test pulse). Peak I_Na_ measured in the test pulse was normalized to peak I_Na_ during the conditioning pulse and plotted as a function of the period between the conditioning and test pulses. These data (i.e. recovery ratio versus time) were fit with a mono-exponential function:

$$f(t)=1-A\;e^{-t/\tau }$$ (4)

where τ is the recovery time constant, A is the amplitude of the component (equal to 1 in mono-phase recovery).

It has previously been established [12] that most Na^+^ channel blockers cause the recovery from inactivation kinetics to display a bi-exponential time-course, with the slow recovering component arising from unblock of the channels and the faster component mapping the time course of recovery from inactivation of the unblocked Na^+^ channels. It is noted that the slow time constant estimated from the bi-exponential fit of the recovery from inactivation does not depend on the blocker concentration while the amplitude of the slow recovering component is a direct measure of the number of Na^+^ channels that are blocked by the drug [13, 14]. Using these properties, we estimated the affinity of mexiletine binding to the channels using the following Sigmoidal function:

$$f(x)=\frac{100}{1+10^{\left(x-\text{log}\;EC50\right)}}$$ (5)

where f(x) is the fractional amplitude of the slow recovering component, “x” is the concentration of mexiletine applied and *EC50* is the estimate of mexiletine blocking effectiveness.

### Simulation studies

To investigate the effects of *SCN5A* mutations on human ventricular myocyte electrophysiology, we modeled their Na^+^ channel behavior using the O’Hara-Virag-Varro-Rudy model (OVVR) [15] and the voltage clamp data (see Results). OVVR Na^+^ channel model parameters were iteratively adjusted one-by-one until the average absolute difference between experimental and simulation data was <0.1%. The parameters used for the simulation study can be found in the Results and were determined in the following order: Maximal G_Na_ scaled according to the ratios in experimental data (normalized with respect to WT) since human atrial and CHO-K1 cells do not have equivalent G_Na_; V_1/2_ and k for m_∞_ adjusted to match steady-state activation in experimental data; V_1/2_ and k for h_∞_ adjusted to match steady-state inactivation in experimental data; Sτ_j_ adjusted to match recovery from inactivation in experimental data; Sτ_h_ adjusted to match I_Na_ decay at 50% in experimental data; and maximal G_NaL_ scaled to match the ratios in experimental data (see Results).

All voltage clamping protocols applied to the modified OVVR models were identical to those in the experimental study. An S1S2 pacing protocol was applied to each OVVR model parameter set (see Results) to investigate the effects of the *SCN5A* mutations on action potential dynamics. Each OVVR model was preconditioned by basal pacing at a cycle length (CL) of 700ms for 100 beats using a 2ms-long stimulus at twice capture amplitude. Single S2 stimuli were then applied beginning at a CL of 690ms, and decreased by 10ms decrements until loss of stimulus capture. Action potential duration (APD) at 90% repolarization was computed for each S2 beat according to Bayer *et al* [16], in addition to the maximum change in membrane potential (dV_m_/dt) during action potential upstroke, action potential activation times at the 0mV threshold crossing of the action potential upstroke, and peak whole-cell I_Na_ (early and late) by summing all Na^+^ dependent currents in the OVVR model. Each was plotted against the S2 diastolic interval (DI).

Single-cell voltage clamping and pacing simulations were performed using the Cardiac Arrhythmia Research Package CARP [17] running on a single CPU of a generic desktop computer. A time step and temporal output of 20μs was used for all simulations in order to sufficiently capture rapidly changing I_Na_ behavior.

### Statistical analysis

Pclamp 8.2 and Clampfit 10 (Molecular device, USA), Excel (Microsoft, USA) and Prism 5.3 (Graphpadsoftware, CA, USA) were used for data acquisition and analysis. Data are presented as mean±standard error. Unpaired student *t test* and one-way ANOVA followed by Tukey test were used to compare the means. Statistical significance was considered when *p*<0.05.

## Results

### Clinical profile

**Fig 1:**
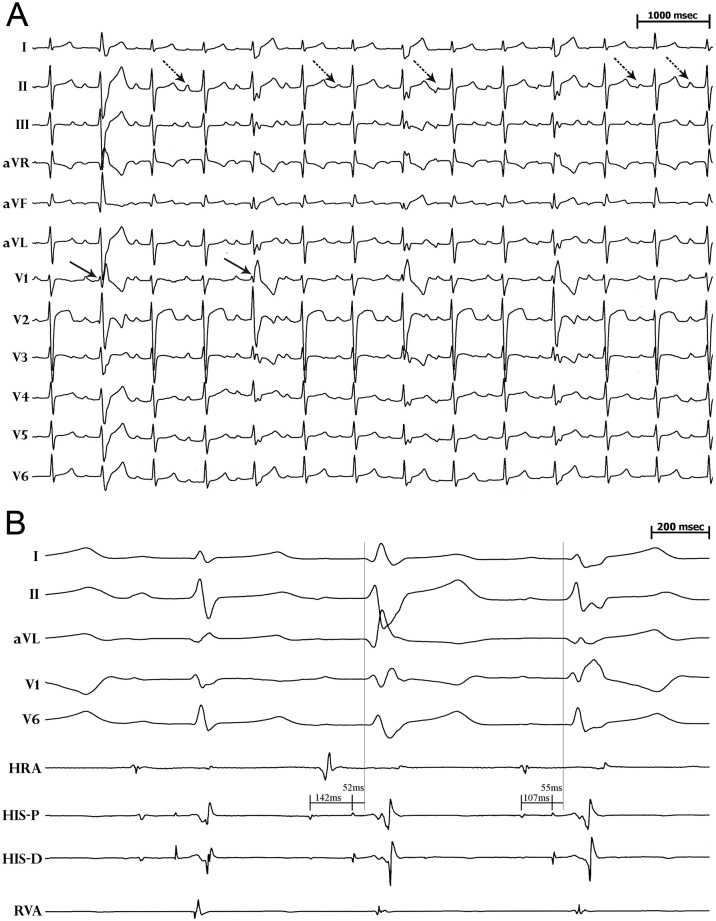
ECG and intracardiac recordings. **A.** Resting 12-lead ECG showing sinus tachycardia, mild QTc prolongation (470ms), atypical ST elevation in lead V2, rate-independent varying QRS aberrancy (solid arrow), and rate-independent changes in P wave morphology (dashed arrow). **B.** Surface ECG and intracardiac electrograms during sinus rhythm showing varying QRS aberrancy and normal HV interval of 55ms. Abbreviations: surface ECG leads I, II, aVL, V1, V2; bipolar electrograms from high right atrium (HRA), His bundle-proximal (HIS-P), His bundle-distal (HIS-D), right ventricular apex (RVA).

The proband is a 22-year old asymptomatic male, with a structurally normal heart and no family history of sudden death, who presented with an abnormal resting 12-lead ECG showing sinus tachycardia at 105bpm, mild QTc prolongation (420-470ms) and atypical ST elevation in V2 and V3. Striking beat-to-beat variation in P wave morphology and RBBB-aberrancy were seen in the absence of premature ectopy or heart rate acceleration (Fig 1A). Invasive electrophysiology testing revealed normal atrio-ventricular (AV) nodal and His-Purkinje conduction, based on normal atrio-Hisian (AH) interval, AV nodal effective refractory period (ERP) and His-ventricular (HV) interval (Fig 1B).

**Fig 2:**
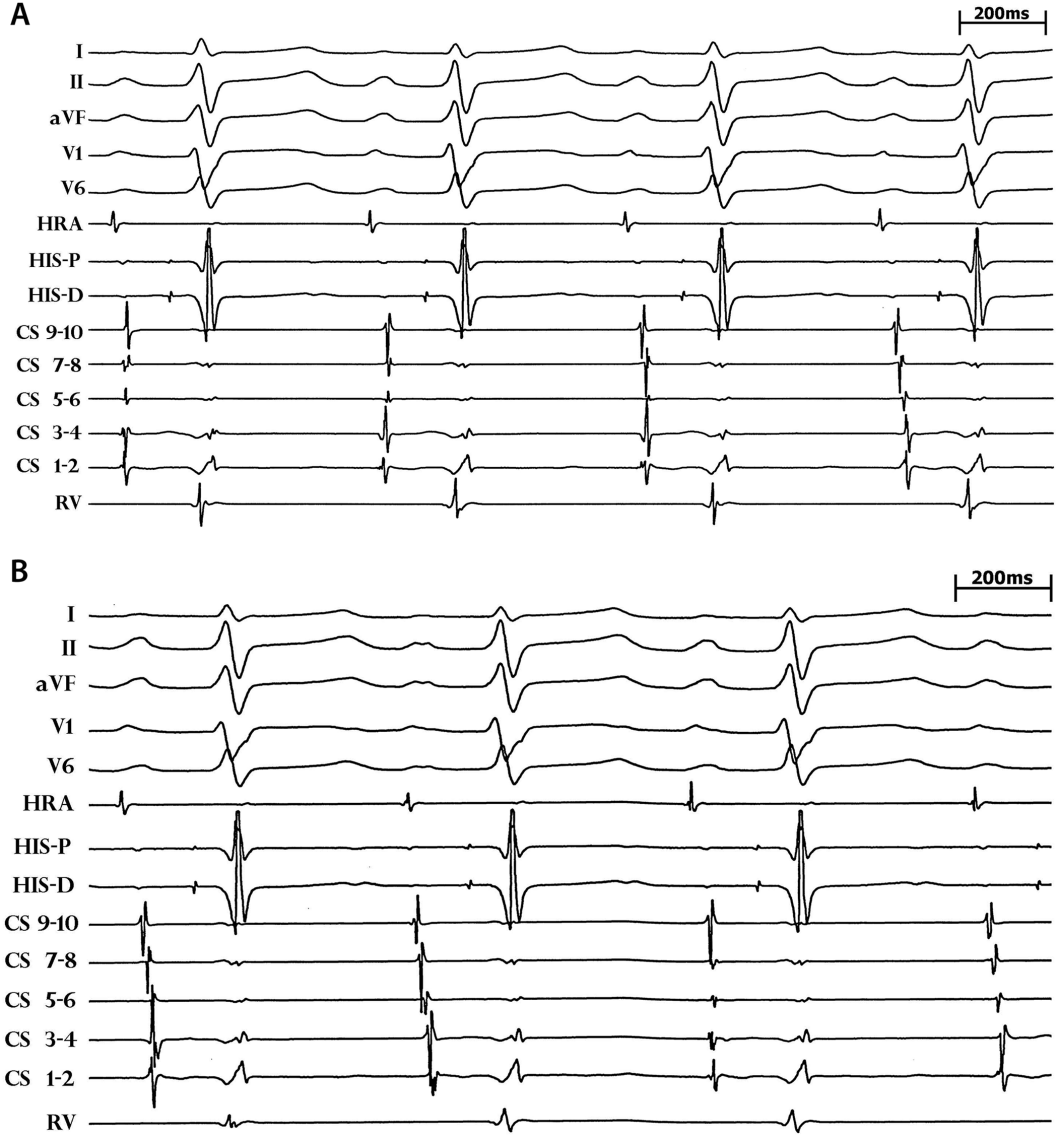
Varying atrial activation in sinus rhythm. **A.** Surface ECG and intracardiac electrograms showing varying P wave morphology and CS activation during sinus rhythm without changing heart rate. Note CS activation is distal to proximal on the first two beats, but then reverses by the fourth beat. **B.** Surface ECG and intracardiac electrograms showing varying P wave morphology and right atrial activation during sinus rhythm without a change in heart rate. Note the difference in high right atrial to His bundle activation time between the first and second beats. Abbreviations are the same as in Fig 1. CS indicates bipolar electrograms recorded from the proximal CS (CS 9–10) to distal CS (CS 1–2).

Atrial activation in sinus rhythm gradually changed along the left atrium-coronary sinus (CS) (CS_distal_→CS_proximal_ transitioning to CS_proximal_→CS_distal_) despite constant heart rate (Fig 2A). Spontaneous changes in right atrial activation also occurred as manifested by varying high right atrial to His bundle activation times without a change in heart rate. These spontaneous abnormal shifts in right and left atrial activation were accompanied by alterations in surface P wave morphology (Fig 2B).

**Fig 3:**
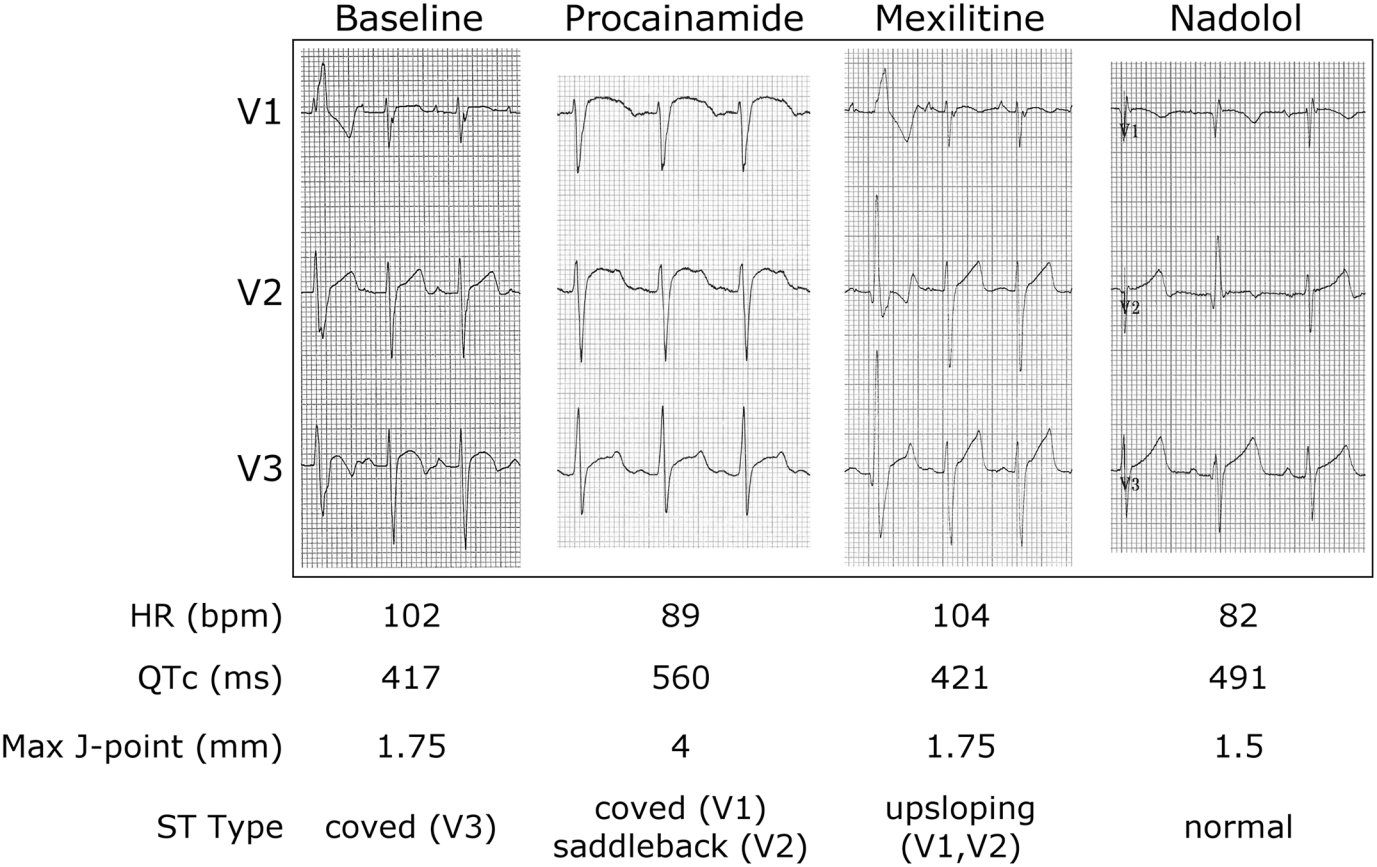
ECG response to drug intervention. Baseline resting ECG shows sinus tachycardia, normal QTc interval, atypical ST elevation in V3, and one right bundle branch block (RBBB) aberrant sinus beat. Procainamide resulted in sinus slowing, marked ST elevation in V1 and V2, but no Brugada type 1 ECG pattern. Mexilitine did not change the resting sinus rate, nor shorten the QTc interval. QRS aberrancy in one sinus beat is still present. Nadolol produced sinus slowing without marked QTc prolongation (i.e. >500 ms).

Mexiletine therapy (100mg twice daily) produced no change in P wave or QRS morphology, no appreciable change in the QTc interval or ST segments, and no effect on resting sinus rate. Mixed Na^+^ and K^+^ channel blockade with procainamide (10mg/kg infused at 50mg/min) did not provoke a Brugada type 1 ECG pattern, although interpretation was confounded by the baseline QRS aberrancy and atypical ST elevation in the anterior precordial leads. Beta-blocker challenge with nadolol (80mg daily) produced sinus slowing without marked QTc prolongation (i.e. >500 ms) (Fig 3).

### Genetic testing

**Fig 4:**
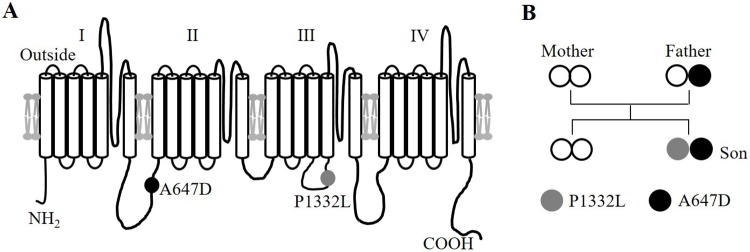
Location of A647D and P1332L mutation. **A.** Schematic representation of the membrane topology of *SCN5A* and the location of the A647D and P1332L mutations. The Na^+^ channel consists of 4 homologous domains (labelled I to IV), each with 6 transmembrane segments. **B.** Pedigree of patient’s family. The proband is the son with both the P1332L and A647D *SCN5A* mutations.

Genetic testing of the proband revealed two heterozygous, missense mutations in *SCN5A*, A647D and P1332L. The P1332L mutation was *de novo*, but the A647D mutation was inherited from the father, who manifested an asymptomatic type 3 Brugada ECG pattern. No mutations were identified in the proband’s mother and only sibling, both of whom had normal ECGs (Fig 4).

Cis/trans genotyping of the proband’s compound mutation was evaluated as follows: First, the family’s genotype was considered, but cis/trans could not be clarified because one mutation was *de novo* and the proband had no offspring. Next, cis/trans status was assessed using reverse-transcriptase PCR and amplification of cDNA from isolated RNA from the proband’s white blood cells. TA cloning of cDNA PCR products then allows for direct sequencing of specific alleles. We attempted this for *SCN5A* and used KCNQ1 as a control from our proband. Although our protocol generating *KCNQ1* cDNA, *SCN5A* cDNA could not be generated, which is somewhat expected since there is essentially no *SCN5A* expression in white blood cells [18, 19]. In absence of data establishing whether our mutations were present in the *cis* versus *trans* configuration, we considered both possibilities in our heterologous expression studies. In particular, we compared the I_Na_ properties of A647D-P1332L *SCN5A* channels (i.e. *cis*) with the mixture of P1332L *SCN5A* channels and A647D *SCN5A* channels coexpressed (i.e. *trans*, P1332L+A647D).

### Peak Na^+^ currents and steady-state activation

**Fig 5:**
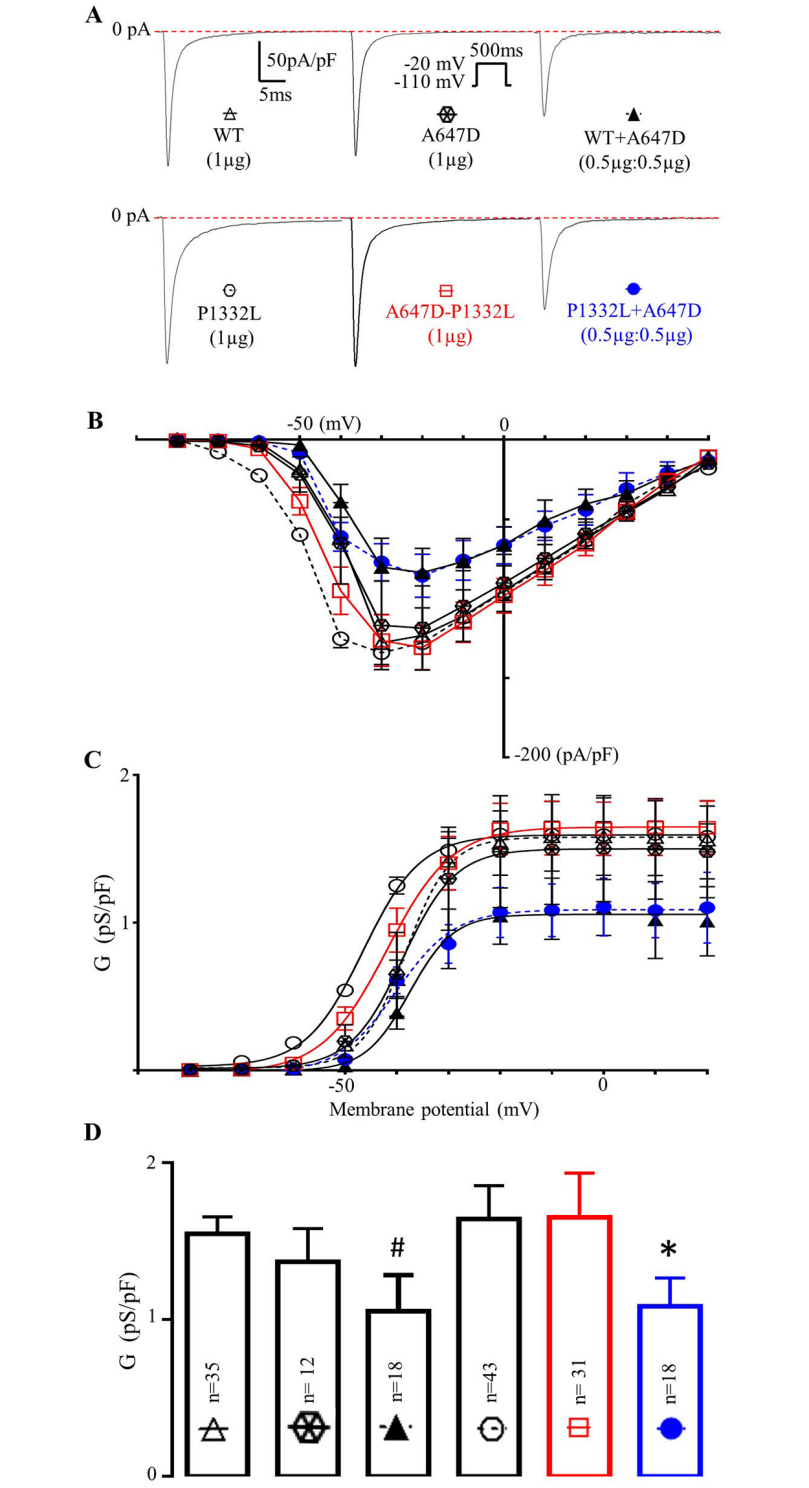
A647D mutation decreases I_Na_ of WT and P1332L when co-expressed. **A.** Typical whole-cell traces of I_Na_ recorded in CHO-K1 cells expressing WT or P1332L channels with or without A647D channels. **B.** Summary of peak I_Na_ density measurements as a function of voltage in CHO-K1 cells (n = 7 in each group). **C.** Summary of the whole-cell Na^+^ channel conductance (G_Na_) as a function of voltage with the lines showing the best fits to a Boltzmann function (n = 7 in each group). **D.** Comparison of G_max_ (estimated by Boltzmann fits to the data in Panel C) for the indicated channels expressed in CHO-K1 cells. #: P = 0.036 versus WT alone; *: P = 0.039 versus P1332L alone.

Since previous studies have established that A647D is linked to Brugada syndrome, in association with reduced current densities when co-expressed with WT in heterozygous system [5], we hypothesized initially that the A647D and P1332L mutations were present in our proband in a *trans* configuration, thereby mitigating the QT prolongation induced by the P1332L mutation as a result of A647D-dependent reductions in the late I_Na_ currents generated by P1332L channels. Fig 5 confirms previous studies [5], showing that co-expression of A647D and WT *SCN5A* channels reduced peak I_Na_ densities in CHO-K1 cells compared to either WT channels or A647D *SCN5A* channels alone. Specifically, Fig 5A shows typical raw I_Na_ traces from WT, A647D alone and WT+A647D channels, recorded in response to depolarizing steps to -20mV from a holding potential of -110mV. Fig 5B shows the mean peak I_Na_ densities as a function of voltage and establishes that peak I_Na_ is reduced when WT and A647D channels are co-expressed (in equal amounts). To more accurately quantify the ability of A647D to reduce peak I_Na_ densities, we estimated the I_Na_ conductance (i.e. G_Na_(V_m_)), normalized to cell capacitance, as a function of V_m_. Fitting G(V_m_) to the Boltzmann equation revealed that the G_max_ of A647D alone (1.37±0.21pS/pF) was similar (P = 0.48) to WT (1.58±0.10pS/pF); but that G_max_ was significantly reduced (P = 0.036) when A467D was co-expressed with WT Na^+^ channels (1.05±0.22pS/pF) (Fig 5C). Having validated the previous studies by Hoshi et al [5], we next explored whether A647D could also reduce I_Na_ generated by P1332L channels.

As summarized in Fig 5B, co-expression of A647D with P1332L reduced peak I_Na_ at all the voltages studied compared to P1332L. G_max_ calculated in CHO-K1 cells expressing P1332L channels alone was 1.64±0.21pS/pF and was reduced (P = 0.039) to 1.08±0.17pS/pF when A647D and P1332L were co-expressed. The magnitude of reduction was similar to that seen when A647D and WT channels were co-expressed. In contrast, G_max_ was not decreased in the *cis* construct, A647D-P1332L, (1.65±0.28pS/pF) compared to WT. Taken together, these results are consistent with our hypothesis that A647D and P1332L, in the *trans* configuration, can lead to reduced peak I_Na;_ thereby explaining the conduction abnormalities seen in our patient.

### Inactivation properties and late Na^+^ currents

**Fig 6:**
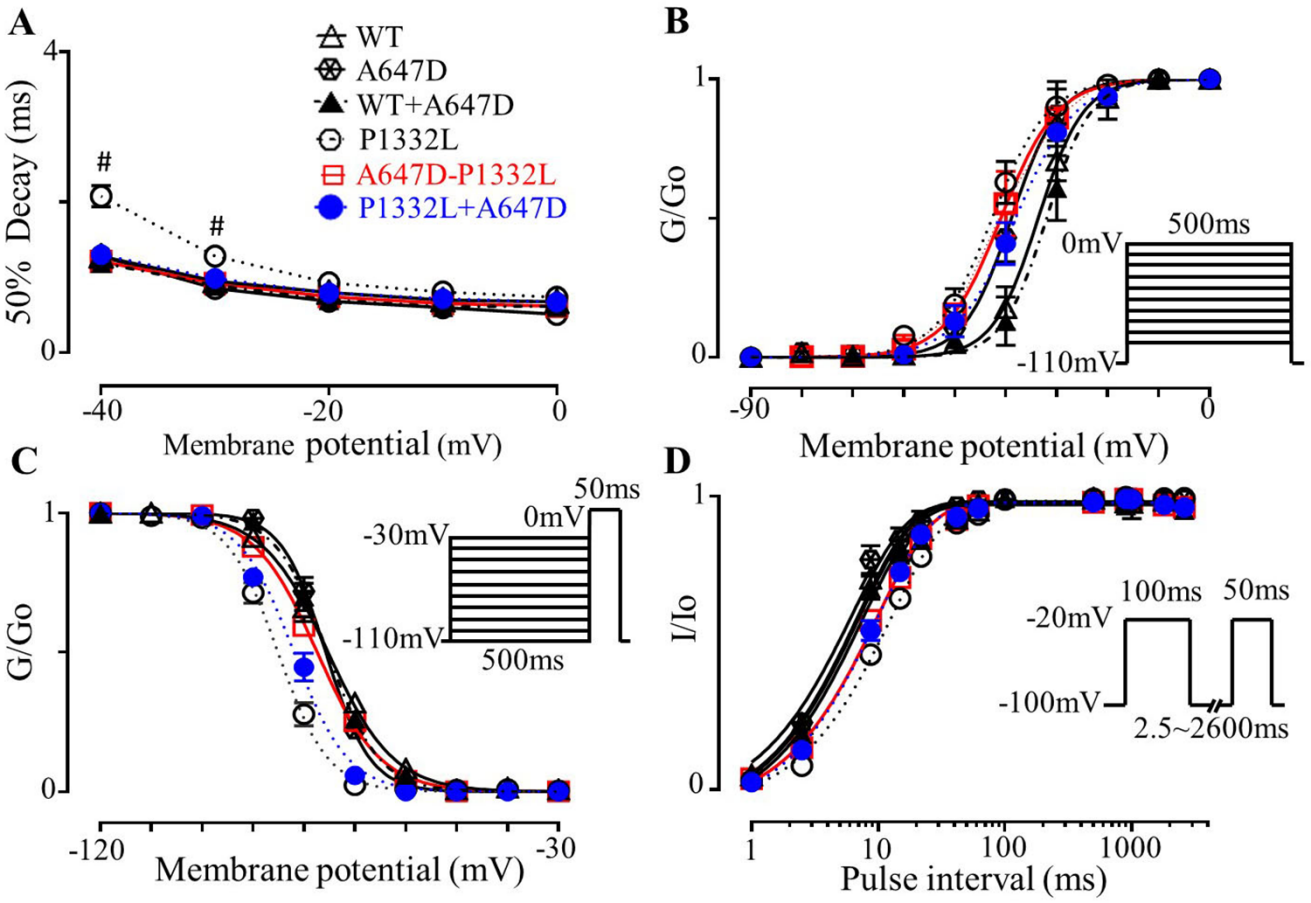
Biophysical features of I_Na_ in CHO-K1 over-expressed with WT, P1332L and co-expressed with A647D. **A.** The time for 50% decay of I_Na_ measured from its peak as a function of membrane voltage. **B.** The whole-cell Na^+^ channel conductance is plotted as a function of the membrane potential for the voltage protocols shown. Conductance was estimated as described in the Methods. The lines were generated from a non-linear least-squares fit to the Boltzmann equation and the parameters for the voltage for 50% activation and the slope factor are summarized in Table 2. **C.** Steady-state inactivation curve, whole-cell Na^+^ channel conductance; the 50% inactivation and slope are listed in Table 2. **D.** I_Na_ recovery from inactivation curve; the recovery time constants and sample size are listed in Table 2.

**Fig 7:**
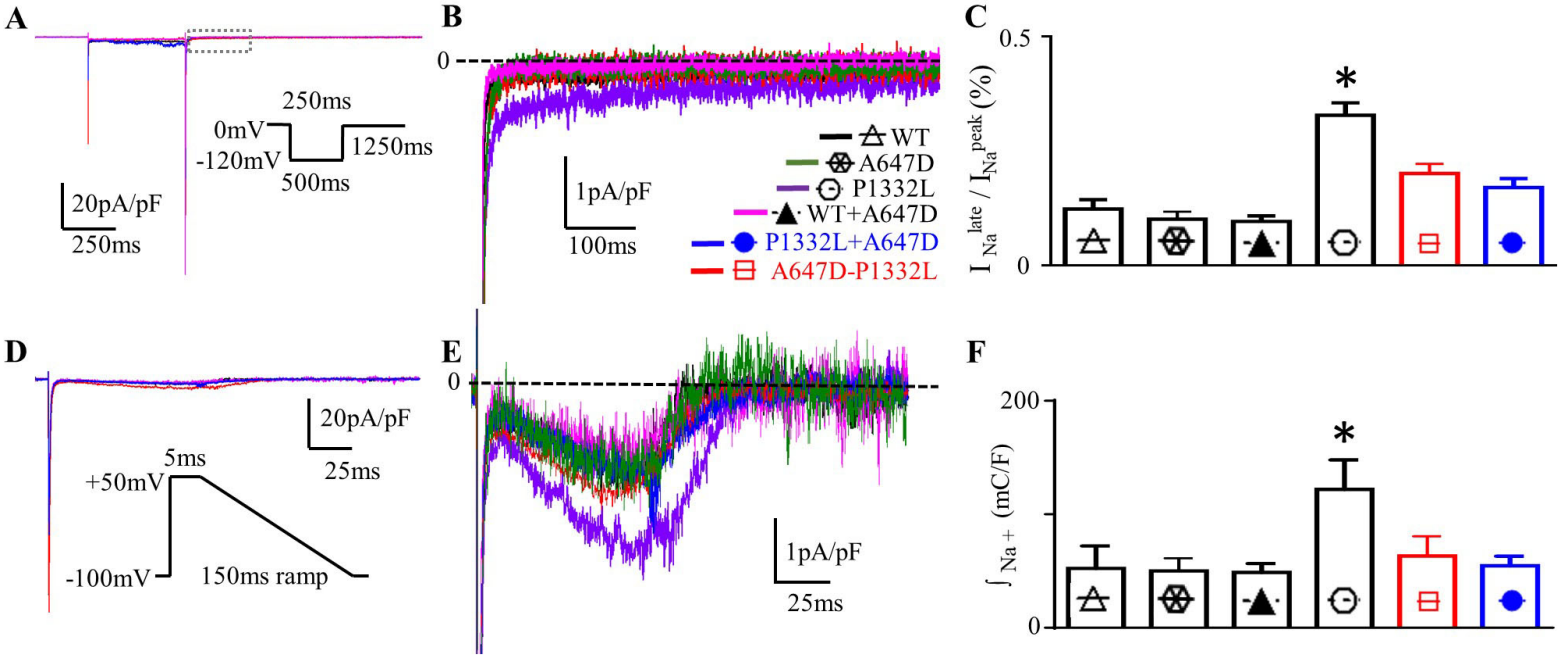
Effects of A647D channels on late currents. **A.** Typical current traces normalized for membrane capacitance recorded from CHO-K1 expressed with WT or P1332L channels with or without A647D channels. **B.** Same currents (as in A) beginning after the peak I_Na_ at a higher magnification, late I_Na_. **C.** Summarizes the ratio of I_Na_ measured at 150ms to peak I_Na_ (*: P<0.05 for P1332L (n = 14) compared to WT (n = 8), A647D (n = 5), WT+A647D (n = 7), A647D-P1334L (n = 7) and P1332L+A647D (n = 10)). **D.** Typical traces normalized for cell capacitance recorded with voltage protocol in the inset. **E.** The same traces (as shown in D) displayed at a higher current amplitude resolution to more clearly illustrate late I_Na_. The broken line represents the zero current level with all currents being leak-corrected. **F.** Summarized the integral of absolute (not normalized) inward Na^+^ charge movement during the ramp protocol (*: P<0.05 for P1332L (n = 6) when compared to WT (n = 6), A647D (n = 5), WT+A647D (n = 6), A647D-P1332L (n = 6) and P1332L+A647D (n = 9)).

We next sought to assess the impact of A647D on late I_Na_ generated by P1332L by considering both *cis* versus *trans* arrangement of the mutations, in order to explain the mild QTc prolongation in our patient. Consistent with previous studies [4], P1332L channels displayed pronounced slowing of I_Na_ current decay compared to WT channels. Specifically, the time required for 50% decay of I_Na_ at -30mV was prolonged (P = 0.021) in P1332L channels (1.28±0.11ms, n = 8) compared to WT channels (0.94±0.06ms, n = 8) as summarized in Fig 6A. Moreover, fitting the time course of I_Na_ decay (see Methods) revealed that the slowest decay component was prolonged in P1332L channels compared to WT channels (Table 1). This slowing of I_Na_ decay in P1332L channels was associated, as expected, with a marked increase in the magnitude of I_Na_ measured 150ms after initiation of depolarizing steps to 0mV (late I_Na_). Indeed, late I_Na_ / peak I_Na_ was 0.32±0.03% (n = 14) in P1332L channels which was almost 3-fold larger (P<0.0001) than 0.12±0.02% (n = 8) in WT channels (Figs 7A–7C).

**Table 1:** Comparison of inactivation kinetics of I_Na_ in response to step depolarizations.

	WT	A647D	WT+A647D	P1332L	A647D-P1332L	P1332L+A647D
A1 (%)	85.91±2.90	80.45±2.82	81.21±2.71	86.57±1.63	81.00±2.01	86.18±2.75
Tau1 (ms)	0.64±0.06	0.34±0.04	0.38±0.06	0.72±0.11	0.51±0.06	0.49±0.08
A2 (%)	12.71±2.60	18.52±2.11	16.35±1.88	11.79±1.15	17.49±2.16	12.25±2.98
Tau2 (ms)	2.79±0.18	1.81±0.21	1.82±0.27	3.27±0.74	2.05±0.28	2.19±0.34
A3 (%)	1.39±0.32	1.03±0.34	1.69±0.32	1.64±0.53	1.51±0.52	1.56±0.55
Tau3 (ms)	17.64±1.39	11.34±1.49	10.72±1.44	43.73±13.14*	17.83±1.92	14.13±2.02

WT, A647D, P1332L and A647D-P1332L: 1μg; WT+A647D and P1332L+A647D: 0.5μg:0.5μg

*: P<0.01 when compared to all other groups using one-way ANOVA followed by Tukey test

To assess the potential impact of slowed inactivation rates of the I_Na_ generated by P1332L channels, we applied voltage protocols to our CHO-K1 cells that crudely mimic the voltage profile of ventricular action potentials in humans. As can be seen in Figs 7D–7F, P1332L channels displayed much larger leak-subtracted inward I_Na_ during slow ramp repolarizations compared to WT channels. The net inward Na^+^ charge movement estimated by integrating the I_Na_ during the repolarization ramp was 121.9±25.9mC/F (n = 6) for P1332L channels which was more than 2-fold greater (P<0.0001) than that for WT channels (52.2±19.8mC/F, n = 6).

In addition to slowed inactivation rates in P1332L channels, the steady-state activation properties (as measured by V_1/2_) were shifted (P = 0.027) markedly toward negative voltages in P1332L (to -42.02±1.97mV) compared to WT channels (-33.67±2.32mV) (Table 2, Fig 6B). Steady-state inactivation was also shifted (P = 0.036) negatively in P1332L (to -85.10±1.66mV) compared to WT channels (-75.26±0.52mV) (Table 2, Fig 6C) while the rate of recovery from inactivation was slowed (P<0.0001) in P1332L (11.68±0.57ms) compared to WT channels (6.05±0.29ms) (Table 2, Fig 6D). Taken together, these biophysical results collectively indicate that the rate of entry into, and exit from, the inactivation state in P1332L channels was slowed relative WT channels, as reported previously [4].

**Table 2:** Comparison of biophysical parameters.

	Steady-state activation			Steady-state inactivation			Time Constant Inactivation Recovery	
	V_1/2_ (mV)	k (mV)	N	V_1/2_ (mV)	k (mV)	N	τ (ms)	N
WT	-33.67±2.32	4.79±0.32	9	-75.26±0.52	5.94±0.24	9	6.05±0.29	7
A647D	-35.86±2.13	5.06±0.26	6	-75.71±1.01	4.34±0.52	6	6.18±0.30	6
WT+ A647D	-31.67±2.51	4.83±0.27	6	-75.38±0.27	5.09±0.22	8	6.49±0.44	7
P1332	-42.02±1.97*	5.24±0.51	8	-85.10±1.66*	4.95±0.22	8	11.68±0.57*	7
A647D-P1332L	-38.95±2.33	5.13±0.47	6	-78.38±0.99	5.12±0.62	6	9.87±0.51	6
P1332L+A647D	-36.72±2.44	5.64±0.40	7	-81.97±1.59	5.31±0.92	7	9.21±0.64	9

WT, A647D, P1332L and A647D-P1332L: 1μg; WT+A647D and P1332L+A647D: 0.5μg:0.5μg

*: P<0.05, compared to all other groups using one-way ANOVA followed by Tukey test

In order to assess the consequences of the *trans* configuration of the A647D and P1332L mutations on late I_Na,_ the late I_Na_/ peak I_Na_ ratio was measured at 150ms after depolarizing steps to 0mV. As shown in Fig 7C, the late I_Na_ / peak I_Na_ ratio was smaller (P = 0.0003) when A647D and P1332L channels were coexpressed (0.17±0.02%, n = 10) compared to P1332L channels alone (0.32±0.02%, n = 14). These reductions were associated with less (P = 0.0099) net inward Na^+^ charge entry in co-expressed A647D+P1332L channels (54.57±8.65mC/F, n = 9) versus P1332L channels alone (121.90±25.91mC/F, n = 5) as summarized in Fig 7F. These effects of A647D channels on the late I_Na_ generated by P1332L were accompanied by small changes in the rate inactivation (Table 1, Fig 6A), steady-state activation properties (Fig 6B), steady-state inactivation properties (Fig 6C) and recovery from inactivation (Fig 6D) to values that were intermediate between values recorded when A647D and P1332L were expressed alone, thereby assisting in reducing the next impact of the P1332L channel on late I_Na_ (Table 2).

By comparison, A647D channels expressed alone or co-expressed with WT had no impact on late I_Na_ at 150ms following depolarizations to 0mV (Fig 7C), the net inward Na^+^ charge movement (Fig 7F), the rate of entry into inactivation (Table 1, Fig 6A), steady-state activation properties (Fig 6B), steady-state inactivation properties (Fig 6C) and rate of the recovery from inactivation (Fig 6D) (Table 2).

On the other hand, A647D-P1332L channels also displayed reductions (P<0.05) in the late I_Na_ / peak I_Na_ ratio (0.20±0.02%) and the net inward Na^+^ charge entry (63.13±17.26mC/F) (Fig 7) in association with alterations (P<0.05) in activation and inactivation properties (Fig 6) compared to P1332L channels, suggesting that the A647D mutation also modulates the impact of the P1332L mutation when the two mutations occur together in the same channel protein. The implications of these observations are discussed further below.

### Mexiletine blockade of Na^+^ currents expressed in CHO-K1 cells

**Fig 8:**
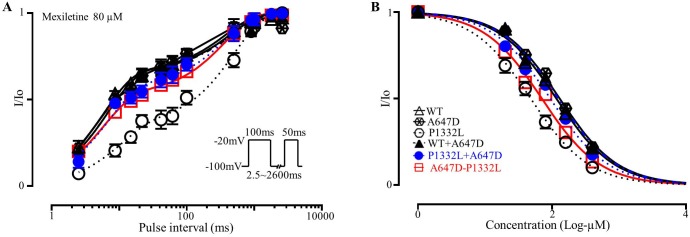
A647D decreased mexiletine sensitivity of P1332L when coexpressed. **A.** The blockage of mexiletine (80μM) on *SCN5A* Na^+^ current was estimated by recovery from inactivation using the protocol shown in the inset with 8 s start-to-start interval. **B.** The dose-response for mexiletine block was determined by plotting the relative amplitude of the slower component of the I_Na_ recovery from inactivation as a function of mexiletine concentration.

As described in the Methods section, the relative blocking efficacy [15] for Na^+^ channel blockers was also assessed by quantifying the recovery from inactivation kinetics of I_Na_ in the presence of mexiletine. As expected [20], mexiletine application resulted in the appearance of a slow, concentration-dependent, kinetic component in I_Na_ recovery from inactivation with a time constant ranging from 301 to 635ms depending on the channel type (Fig 8A). Fitting the recovery from inactivation with bi-exponential functions allows quantification of the fraction of blocked channels from the amplitude of the slowly recovering component [12]. As shown in Fig 8A, in the presence of 80μM mexiletine, the amplitude of the slow recovering component was larger (P<0.0001) in P1332L (0.73±0.03, n = 7) compared to WT (0.35±0.01, n = 6), indicating greater channel blockade. While the co-expression of A647D channels had no effect on the amplitude of this slower recovering component in WT (0.35±0.02, n = 6), it did reduce (P<0.0001) the fraction of channels blocked by mexiletine to 0.45±0.02 (n = 6) when A647D+P1332L were co-expressed. Consistent with these results, Fig 8B shows that the effective concentration required for 50% block of Na^+^ channels by mexiletine was lower (P<0.0001) for P1332L channels (43.29±5.02μM, n = 7) compared to WT (103.61±6.71μM, n = 6), indicating more potent block of P1332L channels relative to WT channels. On the other hand, the blocking efficacy of mexilitine was not different from WT when cells expressed either A647D (117.41±8.32μM, n = 6) or WT+A647D channels (111.73±5.92μM, n = 6). However, the EC50 was shifted to higher (P<0.05) mexilitine concentrations when A647D and P1332L channels were co-expressed (91.65±7.07μM n = 6) compared to P1332L alone.

**Fig 9:**
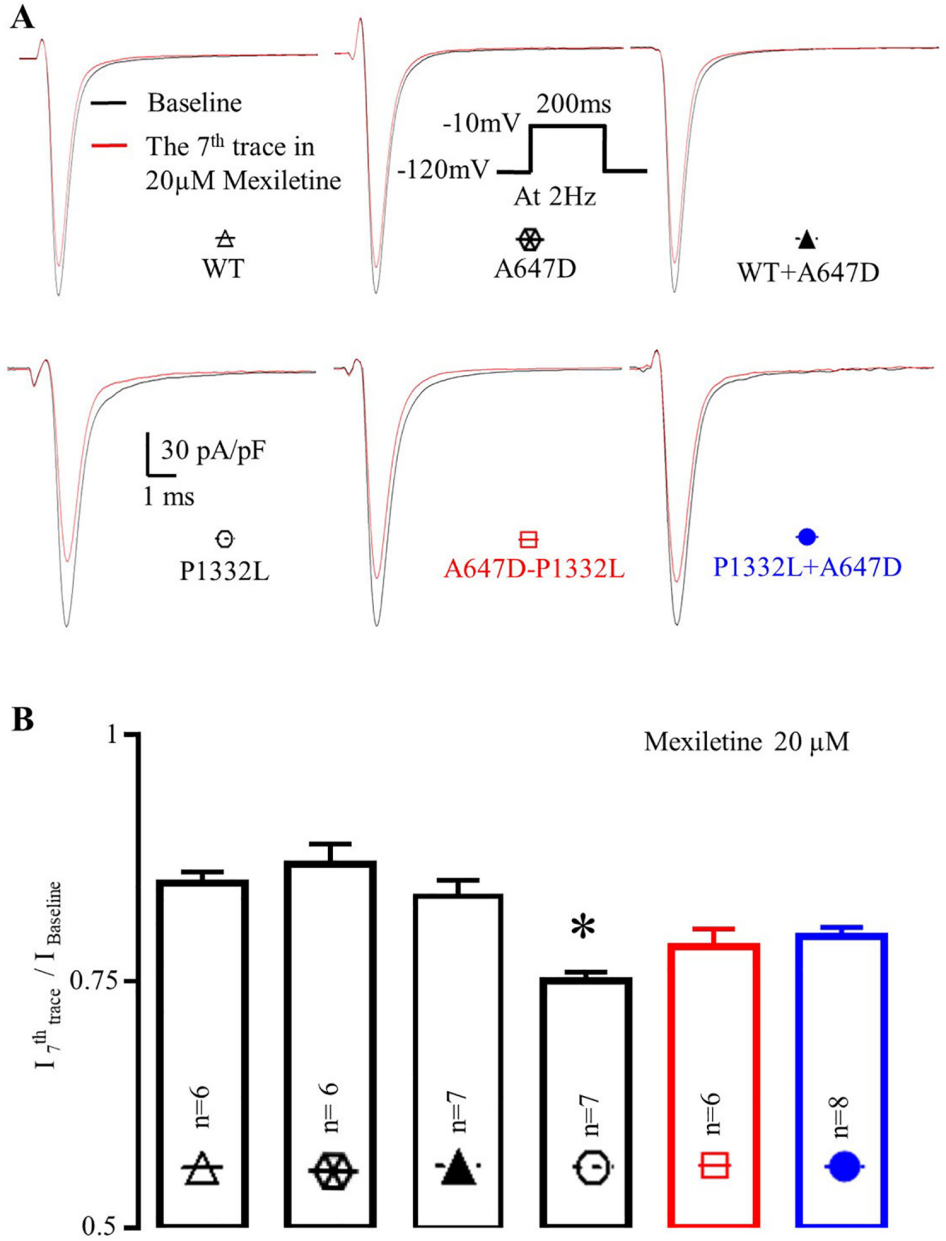
Mexiletine use-dependent block of WT and mutations Na^+^ currents. **A** Normalized representative recordings of I_Na_ in CHO-K1 cells over-expressed with WT and mutant Na^+^ channels using the protocol shown in insets; **B.** Comparison of the ratio of I_Na_^peak^ of the 7^th^ trace in 20μM mexiletine to I_Na_^peak^ of baseline; *: P<0.05, compared to all other groups.

To further characterize the blocking properties of mexiletine in our channels, use-dependence of I_Na_ blockade was also examined. As summarized in Fig 9, the ratio of peak I_Na_ recorded at the 7^th^ pulse to peak I_Na_ in pulse 1 (i.e. peak I_7_/ peak I_1_), recorded when cells were stimulated at 2Hz in the presence of 20μM mexiletine, was 0.75±0.01 (n = 7) in P1332L, which was smaller (P<0.05) than WT channels (0.84±0.02, n = 6) as well as A647D channels (0.86±0.02, n = 6) or WT+A647D co-expressed channels (0.83±0.02, n = 7). Thus, mexilitine showed an increased blocking efficacy for P1332L channels compared to WT, A647D or WT+A647D co-expressed channels. Consistent with the increased EC50 for mexilitine blockade in P1332L+A647D co-expressed channels, the peak I_7_/ peak I_1_ was greater, although not statistically significant, when P1332L+A647D were co-expressed (0.79±0.01, n = 8) compared to P1332L channels alone. Interestingly, A647D-P1332L channels also showed a slightly higher EC50 (70.54±7.18μM, n = 6) compared to P1332L alone and this was matched by a trend towards an increase in peak I_7_/ peak I_1_ (0.78±0.02, n = 6). The implications of this are considered in the Discussion.

### Modelling I_Na_ kinetics and action potential properties

**Fig 10:**
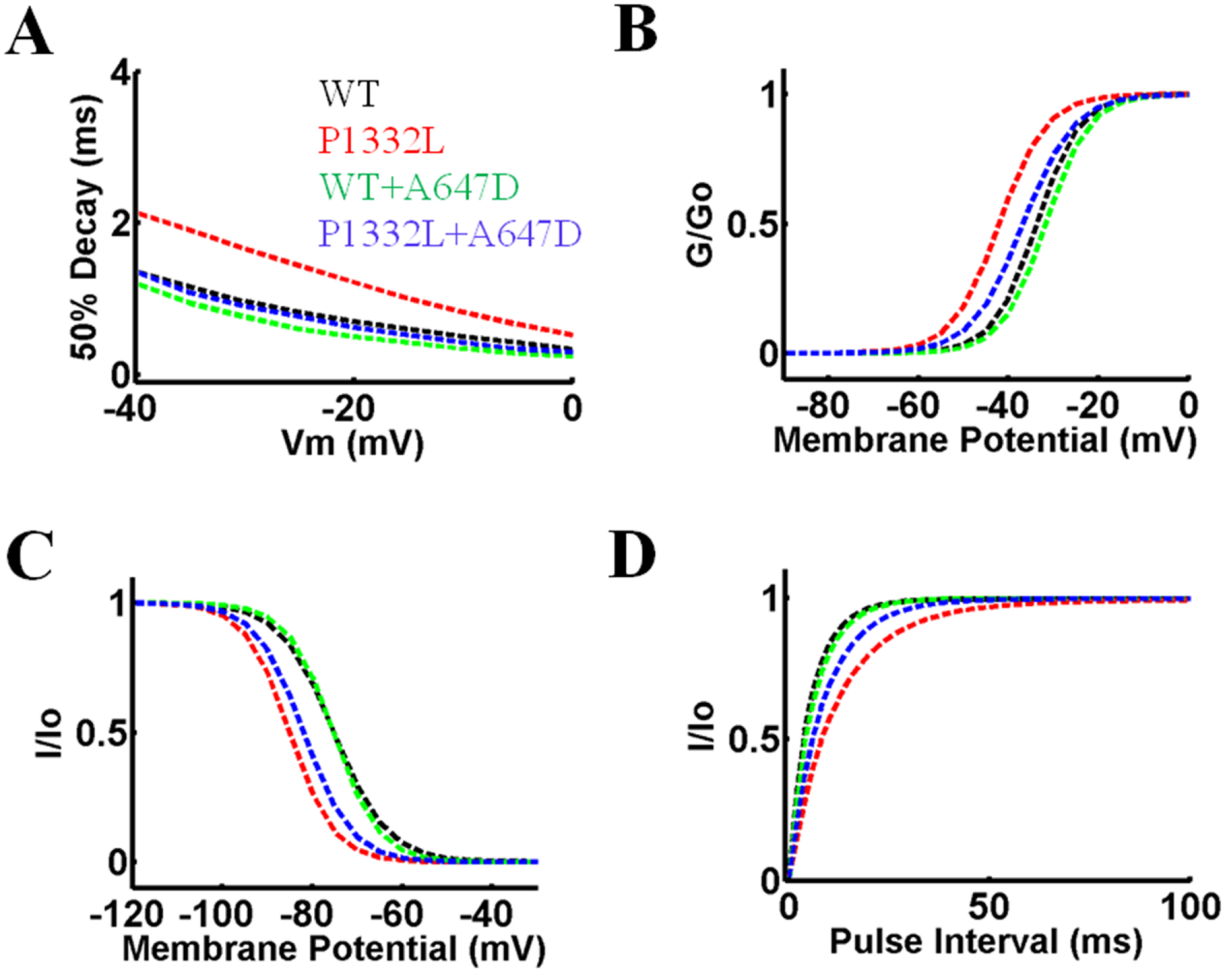
Biophysical features of I_Na_ in the OVVR model simulating WT, P1332L, WT+A647D and P1332L+A647D Na^+^ channels. **A.** 50% peak I_Na_ decay. **B.** Steady-state activation. **C.** Steady-state inactivation**. D.** Recovery from inactivation. In each panel, the kinetics matches the respective experimental data shown in Fig 6.

The OVVR model was implemented using the parameters in Table 3 to simulate WT and mutant human ventricular myocytes based on the experimental data presented in Table 2 and Figs 5–7. I_Na_ kinetics for WT and mutant Na^+^ channels, P1332L, WT+A647D and P1332L+A647D, generated by the model are presented in Fig 10. We did not specifically model the *cis* construct, A647D-P1332L, because its biophysical properties were similar to the *trans* construct, P1332L+A647D, and WT. Moreover, A647D-P1332L did not manifest reduced peak I_Na_, unlike P1332+A647D, which made it less likely to explain our patient’s conduction abnormalities. I_Na_ kinetics of delay, steady-state activation, steady-state inactivation, and recovery from inactivation were matched closely to the respective voltage-clamp data, as shown in Fig 6.

**Table 3:** OVVR model parameters that fit I_Na_ behavior for WT and *SCN5A* mutations.

	WT	P1332L	WT+A647D	P1332L+A647D
G_Na_ (pS/pF)	27750	28800	18440	18970
G_NaL_ (pS/pF)	50	90	40	50
Sτ_j_	0.19	0.59	0.19	0.31
Sτ_h_	2	3.33	1.43	1.82
m_∞_ V_1/2_ (mV)	-41.67	-50.02	-40.67	-46.72
m_∞_ k (mV)	7.79	8.24	6.83	8.64
h_∞_ V_1/2_ (mV)	-70.26	-82.1	-70.38	-77.47
h_∞_ k (mV)	7.94	7.2	7.09	7.31

G_Na_ and G_NaL_ are the maximal conductance for the early and late currents I_Na_ and I_NaL_ in the OVVR model, respectively. The Sτ_j_ and Sτ_h_ are scaling factors for the time constants of the j (second term only) and h (fast and slow) gates in the OVVR model, respectively. In the OVVR model, the steady-state activation (m_∞_) and inactivation (h_∞_ fast and slow) functions were replaced with the following equations: m_∞_(V_m_) = 1.0/(1.0+exp((V_1/2_–V_m_)/k)) and h_∞_(V_m_) = 1.0/(1.0+exp((V_m_-V_1/2_)/k)) using the V_1/2_ and k values listed in the Table above.

**Fig 11:**
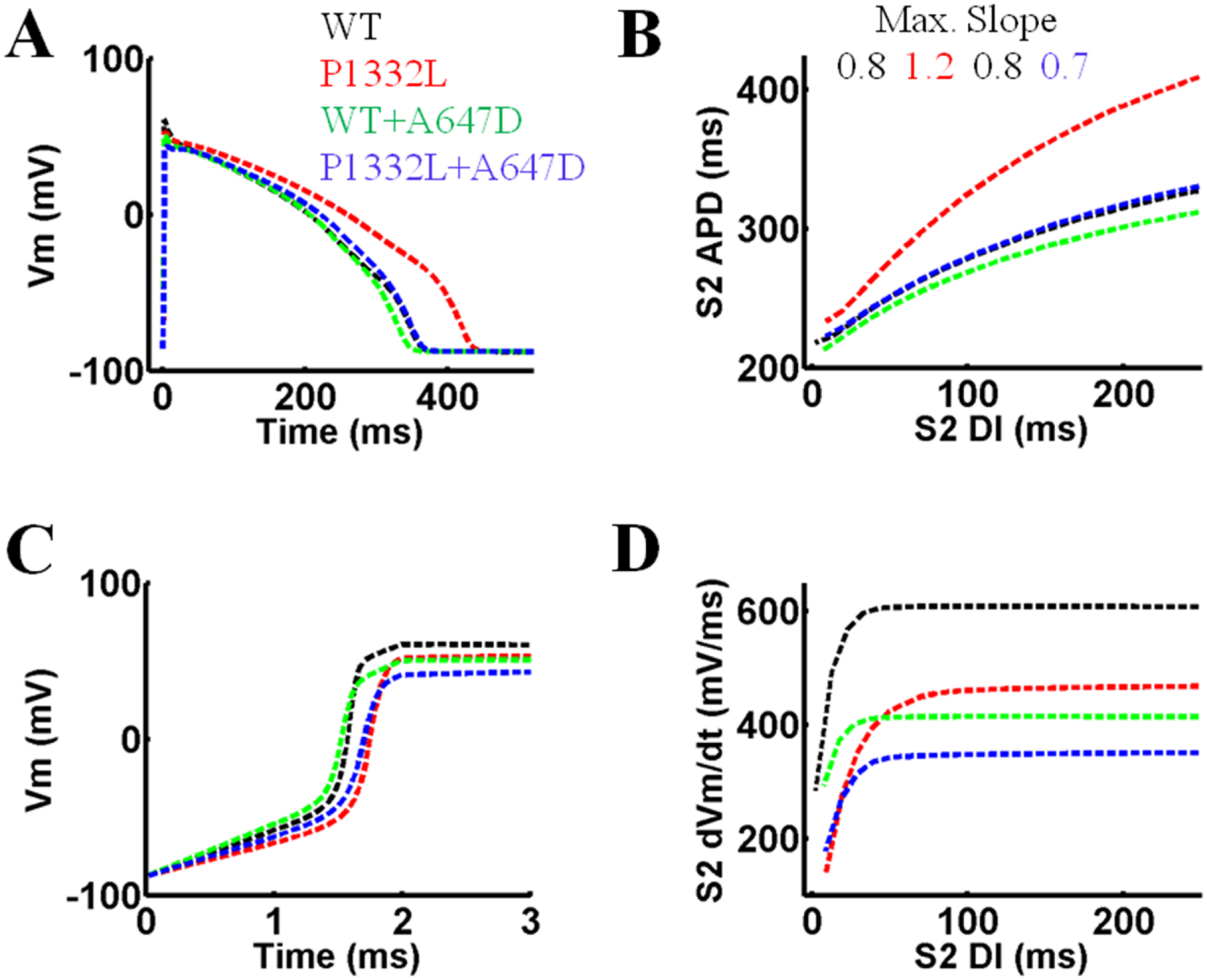
Action potential dynamics in the OVVR model simulating WT, P1332L, WT+A647D and P1332L+A647D Na^+^ channels during S1S2 pacing. **A.** Transmembrane potential for the last S1 beat during S1 pacing. **B.** APD restitution for S2 during the S1S2 pacing protocol with the maximal slope listed for each curve. **C.** Transmembrane potential during the action potential upstroke for the last S1 beat during S1 pacing. **D.** The maximum dV_m_/dt during the action potential upstroke for S2 during the S1S2 pacing protocol.

The dynamic effects of the *SCN5A* mutations on action potential properties were next examined by simulating pacing of ventricular myocytes in the OVVR model using the parameters in Table 3. Myocytes were first paced at a basal CL of 700ms from a resting membrane potential of -80mV. As expected, the APD was 70ms longer for the P1332L model compared to WT, whereas the WT+A647D and P1332L+A647D models had only small effects on APD compared to WT (<15ms) (Fig 11A). With tighter S1S2 coupling intervals during extrastimulus pacing, Fig 11B shows that the APD restitution slope, relative to WT, increased more for P1332 compared to WT+A647D or P1332L+A647D (1.2 vs. 0.7–0.8). Also, during basal pacing at 700ms, the maximum upstroke of the action potential for all three *SCN5A* mutants was less steep than WT (608 vs. 352-469mV/ms) with the lowest slope being observed in the P1332L+A647D model (352mV/ms), as summarized in Fig 11C. Similarly, during extrastimulus pacing with tightly coupled S1S2, the maximal upstroke velocity of the S2 action potential was much less for models with the P1332L mutant compared to WT (285 vs. 142-178mV/ms) (Fig 11D). Of note, the resting membrane potential during pacing did not change more than 1mV between WT and the various *SCN5A* mutants.

### Simulated early and late I_Na_ during pacing

**Fig 12:**
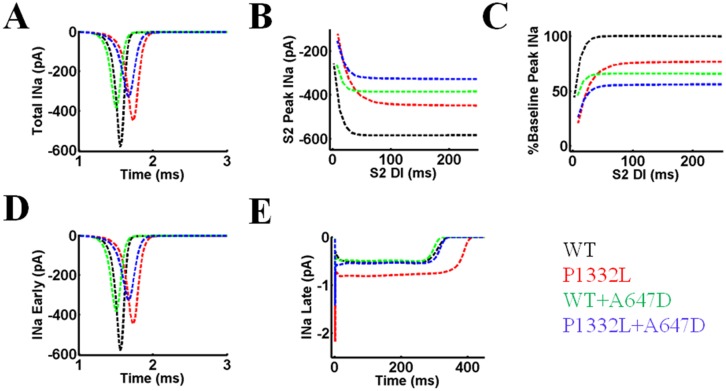
Whole-cell I_Na_ in the OVVR model simulating WT, P1332L, WT+A647D and P1332L+A647D Na^+^ channels during S1S2 pacing. **A.** Whole-cell I_Na_ for the last S1 beat during S1 pacing. **B.** Rate-dependent peak I_Na_ for each S2 in the S1S2 pacing protocol. **C.** The percentage of the baseline WT peak I_Na_ during S1 pacing for each S2 in the S1S2 pacing protocol for the four models. **D.** Early component of I_Na_ for the last S1 beat during S1 pacing. **E.** Late component of I_Na_ for the last S1 beat during S1 pacing.

To assess the underlying basis for the changes in action potential properties seen with the different *SCN5A* channels, we also quantified I_Na_ during action potentials generated from simulated pacing. During basal pacing at 700ms, peak I_Na_ was reduced for P1332, WT+A647D and P1332L+A647D compared to WT, with the greatest reduction seen in P1332L+A647D (Fig 12A). In accordance with action potential upstroke behavior shown in Fig 11D, only constructs with the P1332L mutation significantly reduced peak I_Na_ compared to WT at tighter S1S2 coupling intervals (-121-154 vs. -258pA) as summarized in Fig 12B. When compared to the baseline WT peak I_Na_ during basal pacing at 700ms, the reduction in peak I_Na_ was P1332L 23%, WT+A647D 34%, and P1332L+A647D 44% (Fig 12C). Notably, the early component of I_Na_ during basal pacing was lowest for P1332L+A647D (Fig 12D), while its late component (late I_Na_) was similar to that of WT (Fig 12E).

**Fig 13:**
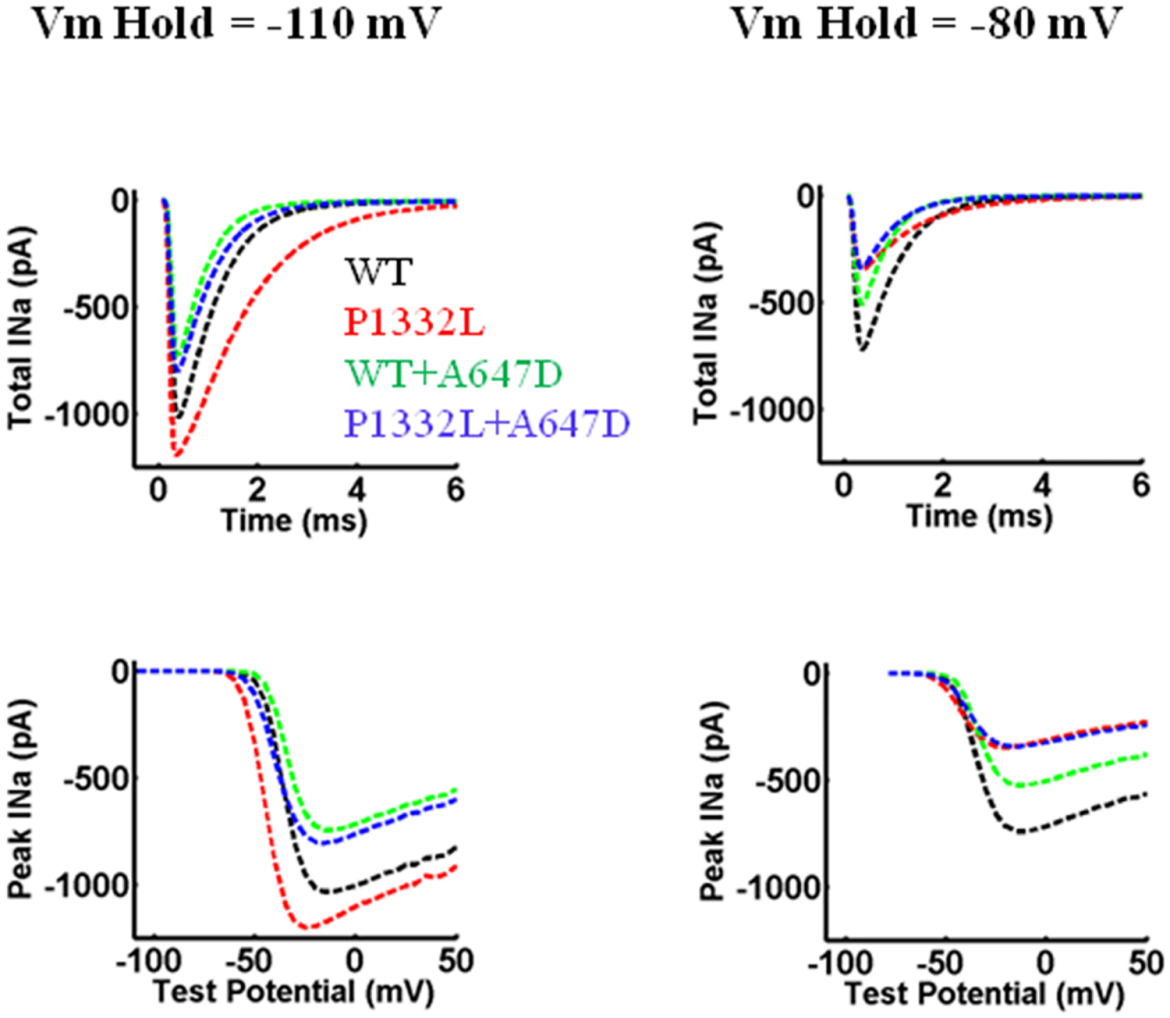
Whole-cell I_Na_ in the OVVR model simulating WT, P1332L, WT+A647D and P1332L+A647D Na^+^ channels during test potentials (V_t_) from a holding potential of -110 mV versus -80 mV. The plots in the top row are whole-cell I_Na_ for V_t_ -20 mV. The plots in the bottom row are peak I_Na_ computed for all V_t_ from -110 to 50 mV.

Interestingly, the lower peak I_Na_ in P1332L+A647D compared to WT+A647D in our simulations was not seen in the experimental studies (i.e. Fig 5B). We suspected that these differences between simulation and experimental results arose from the differences in holding potential, particular since P1332L+A647D show a greater hyperpolarizing shift in steady state inactivation compared to WT+A647D. To test this notion, we repeated our simulation by forcing a resting membrane potential of -110mV as used in our experimental recordings. As shown in Fig 13, when a holding potential of -110mV was used, peak I_Na_ no longer differed between P1332L+A647D versus WT+A647D, consistent with our experimental results.

## Discussion

While the P1332L and A647D *SCN5A* mutations have been individually reported, we describe for the first time the clinical presentation and biophysical properties of the compound mutations, which produced a novel, pleotrophic phenotype of resting sinus tachycardia, mild QTc prolongation, atypical anterior ST elevation and varying P wave and QRS aberrancy. Intracardiac recordings confirmed atrial and ventricular conduction abnormalities based on spontaneous rate-independent changes in atrial activation and RBBB aberrancy. Individually, the P1332L mutation causes a lethal form of LQT3 [4], while the A647D mutation is associated with an atypical form of Brugada syndrome [5]. Neither has been linked to conduction disease. Our proband’s benign clinical presentation and mixed phenotype (i.e. mild QT prolongation and conduction defects) suggested an interaction between these mutations. In support of this, our cellular studies revealed foremost that when A647D and P1332L channels are co-expressed both the peak and late I_Na_ are reduced compared to P1332L channels alone, which, assuming a *trans* presentation of the mutations, may explain the clinical pheotype of our patient. It should be mentioned, however, that late I_Na_ was also reduced in channels harbouring both the A647D and P1332L mutations (i.e. A647D-P1332L). Thus, it is conceivable that our patient possesses the compound mutations in a *cis* configuation. Unfortunately, without the ability to perform direct cis/trans genotyping, the finding of reduced peak I_Na_ with the *trans* construct likely supports the *trans* genotype in our proband.

The presence of the A647D channels also reduced the mexiletine sensitivity observed in the P1332L channels, whether in the *cis* or *trans* configuration. These results explain the mild LQT3 phenotype and the inability of mexiletine to appreciably shorten our probands’s QTc interval, despite the high sensitivity of P1332L channels to block by this drug [4]. Our simulation studies confirmed shorter APD in P1332L+A647D compared to P1332L, and further demonstrated lower peak I_Na_ in P1332L+A647D than P1332L or WT+A647D, which explains our proband’s mild QTc prolongation and conduction defect, respectively. Thus, although mexiletine is reported to be therapeutic in LQT3 patients possessing the P1332L mutation, long-term treatment with this agent was not considered in our proband because it would be ineffective and potentially exacerbate the inherent conduction abnormalities [5].

### Cellular and drug studies explain mild LQTS

Our studies confirmed that the P1332L LQT3 mutation, which is located in a highly conserved region of the S4-S5 cytoplasmic, slows inactivation of I_Na_, in association with hyperpolarizing shifts in steady-state activation and inactivation while also increasing block by mexiletine. We further established that P1332L channels have marked elevation in the net charge entry during voltage ramp protocols designed to mimic human ventricular action potentials, which helps explain the profound QT prolongation and the malignant clinical phenotype seen in patients with this mutation [3,4].

Our studies also confirmed that the A647D *SCN5A* mutation, which is located in the intracellular linker between domains I and II (Fig 4A), caused peak I_Na_ reduction without appreciably affecting other biophysical properties of WT channels. According to Hoshi et al [5], the reduced peak I_Na_ with A647D and WT coexpression is due to an induced trafficking defect which affects cell surface expression of both WT and mutant protein. This explains the Brugada syndrome seen in patients harboring the A647D mutation [5] as well as the P wave and QRS aberrancy and the mild QT prolongation in our patient. Indeed, A647D channels, not only reduced late I_Na_ responsible for the virulent QTc prolongation, but also reduced the effects of the P1332L mutation on the slowed recovery from inactivation as well as the hyperpolarizing shift in steady-state inactivation, whose effects on abnormal conduction was uncovered in our simulation studies as discussed below.

The presence of A647D channels also shifted the steady-state inactivation of P1332L channels to more depolarized potentials, thereby reducing mexiltine block [21, 22] which can account for the lack of QTc shortening with mexiletine in our proband.

### Simulation studies explain novel conduction defects

We used an OVVR-based model to further explore the underlying basis for the clinical features of our proband. While the A647D mutant reduce G_Max_ when coexpressed with P1332L and thereby also reduced the large late I_Na_ generated by the P1332L channels, our simulation results revealed additionally that peak I_Na_ was actually lower in P1332L+A647D compared to WT+A647D. This effect can be traced to the prominent hyperpolarizing shift in the steady-state inactivation curve caused by the P1332L mutation which was more pronounced in P1332L+A647D versus WT+A647D. This in turn slowed action potential upstroke velocity in P1332L+A647D during simulated steady pacing, and the effect was further accentuated with extrastimulation, which is theoretically predicted to cause conduction velocity slowing during resting heart rates in our proband [23]. The importance of this mechanism is highlighted by the observation that when the model cell was hyperpolarized, the effects of either the P1332L or P1332L+A647D mutations on peak I_Na_ were much reduced. Thus, our simulation results suggest that the presence of A647D channels imparted a “double hit” in reducing P1332L I_Na_; thereby accentuating the conduction defects in our proband, which would not be seen in patients harboring either mutation alone. This “double hit” was sufficient to cause beat-to-beat variability in atrial and ventricular conduction, but not fixed His-Purkinje or nodal dysfunction.

### Prior studies on compound *SCN5A* mutations

Compound *SCN5A* mutations are uncommon (<5% of patients) [24] and some have been reported to accentuate disease phenotype, as with R34fx/60+R1195H [25] or W156X+R225W [26] or reduce phenotype as seen with the common *SCN5A* polymorphisms H558R [7, 8] or R1193Q [9]. Our report is the first to illustrate the ability of a compound *SCN5A* mutation to mitigate a lethal phenotype (i.e. LQT3), while concurrently introducing a new phenotype (i.e. conduction abnormalities) and modulating pharmacotherapy (i.e. Class IB agents like mexiletine for LQT3). The therapeutic benefit of these agents can be quite variable in single LQT3 mutations, in some cases due to differences in drug-binding affinity [4]. In one reported LQT3 patient, mexiletine caused paradoxical QTc lengthening and proarrhythmic death after it enhanced mutant channel protein trafficking [27]. Hence, caution should be exercised when prescribing Na^+^ channel blockers in LQT3 and our findings draw attention to the potential interaction of compound mutations on drug pharmacology.

### Limitations

Cis/trans genotyping was not possible. While the *trans* genotype was initially assumed and was able to account for the ECG phenotype and lack of response to therapy of our patient, we also noted for the first time that the presence of both the A647D and P1332L mutations in the same protein also altered the biophysical properties in a manner that might explain some of the features in our patient. It is also acknowledged that our proband’s resting sinus tachycardia could not be readily explained, and this is contrary to the sinoatrial nodal disease reported with other Na^+^ channel mutations or LQTS [1]. Resting sinus tachycardia may have contributed to the shortened QTc our proband, as seen with rapid atrial pacing in the treatment of LQTS. However, sinus slowing with a trial of beta-blocker therapy (i.e. nadolol 80mg daily) in our proband did not result in excessively long QTc intervals (over 500ms), as reported in patients with P1332L (Fig 3).

## Conclusions

We demonstrate a novel pleiotropic phenotype arising from the compound *SCN5A* mutation, P1332L and A647D. The lethal LQT3 phenotype of P1332L was mitigated with the Brugada- associated A647D, which negated the previously reported therapeutic benefit of mexiletine. Concurrently, the A647 mutant reduced I_Na_ sufficiently to produce a novel global cardiac conduction defect affecting the atria and ventricles, but not the sinoatrial or AV nodes. These finding highlight the complex, unpredictable biophysical interactions of LQT3-related compound mutations and the relevance of cellular expression studies in clarifying the role of Na^+^ channel blocking pharmacotherapy.

## Abbreviations

AF: atrial fibrillation
APD: action potential duration
AV: atrioventricular
CS: coronary sinus
ERP: effective refractory period
GFP: green fluorescent protein
HV: His-ventricular
I_Na_: whole cell sodium current
OVVR: O’Hara-Virag-Varro-Rudy model
RBBB: right bundle branch block
WT: wild type
